# Associations between Serum Zinc Levels and the Presence of Distal Symmetric Polyneuropathy in Patients with Type 2 Diabetes Mellitus

**DOI:** 10.1002/edm2.70265

**Published:** 2026-07-20

**Authors:** Hosseinali Mohammadi, Firozeh Sajedi, Younes Mohammadi

**Affiliations:** ^1^ Student Research Committee, School of Medicine Hamadan University of Medical Sciences Hamadan Iran; ^2^ Department of Internal Medicine, School of Medicine Hamadan University of Medical Sciences Hamadan Iran; ^3^ Department of Epidemiology, School of Public Health Hamadan University of Medical Sciences Hamadan Iran

**Keywords:** diabetic neuropathy, diabetic polyneuropathy, distal symmetric polyneuropathy, type 2 diabetes mellitus, zinc

## Abstract

**Introduction:**

Distal Symmetric Polyneuropathy Is the Most Common Form of Diabetic Neuropathy and a Major Source of Morbidity in Type 2 Diabetes. Oxidative Stress Plays a Key Role in Its Pathogenesis, and Zinc—An Essential Trace Element Involved in Antioxidant Defence—Has Been Proposed as a Protective Factor. However, Clinical Evidence on the Association Between Serum Zinc Levels and Diabetic Neuropathy Remains Limited and Inconsistent. This Study Examined the Relationship Between Serum Zinc Levels and the Presence of Distal Symmetric Diabetic Polyneuropathy in Type 2 Diabetes.

**Methods:**

This Nested Case–Control Study Was Conducted Between December 2023 and March 2025 at the Internal Medicine Clinic of Shahid Beheshti Hospital in Hamadan, Iran. Ninety Patients With Type 2 Diabetes Were Enrolled, Including 45 With Distal Symmetric Polyneuropathy and 45 Without Neuropathy, Diagnosed According to American Diabetes Association Criteria. Serum Zinc Levels Were Measured Photometrically. Group Comparisons Used Independent t‐Tests and Chi‐Square Tests. Multivariable Logistic Regression Analysis Was Used to Examine the Association Between Serum Zinc Levels and DSPN While Adjusting for Potential Confounders.

**Results:**

Patients With Distal Symmetric Polyneuropathy Had Significantly Lower Serum Zinc Levels Than Those Without (70.6 ± 12 μg/dL vs. 82.2 ± 16.9 μg/dL, *p* < 0.001). In Multivariable Logistic Regression, Zinc Levels Remained Independently Associated With the Presence of Neuropathy; Each One μg/dL Increase Was Associated With a 5.4% Reduction in Odds of Neuropathy.

**Conclusions:**

Lower serum zinc levels were independently associated with the presence of distal symmetric polyneuropathy in type 2 diabetes. This finding suggests that zinc deficiency may contribute to the development of diabetic neuropathy.

## Graphical abstract

Lower serum zinc levels were independently associated with distal symmetric polyneuropathy in patients with type 2 diabetes mellitus. Each 1 μg/dL increase in serum zinc level was associated with a 5.4% reduction in the odds of diabetic neuropathy, suggesting a potential role of zinc deficiency in DSPN pathogenesis.

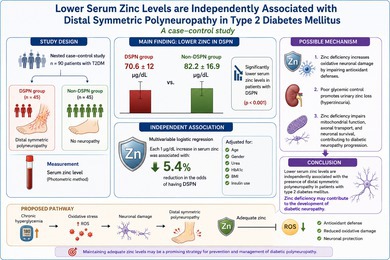

**Abbreviations**
ADA: American Diabetes Association
BMI: Body Mass Index
DPN: Diabetic Polyneuropathy
DSPN: Distal Symmetric Polyneuropathy
eGFR: Estimated Glomerular Filtration Rate
GFR: Glomerular Filtration Rate
HbA1c: Haemoglobin A1c
HDL: High‐Density Lipoprotein
ICC: Intraclass Correlation Coefficient
LDL: Low‐Density Lipoprotein
MNSI: Michigan Neuropathy Screening Instrument
NDS: Neuropathy Disability Score
NSS: Neuropathy Symptom Score
PPI: Proton Pump Inhibitor
ROS: Reactive Oxygen Species
SD: Standard Deviation
SSEP: Somatosensory Evoked Potential
T2DM: Type 2 Diabetes Mellitus
VAS: Visual Analogue Scale

## 1 Background

Diabetes mellitus is a chronic metabolic disorder characterised by persistent hyperglycemia. It is one of the most prevalent endocrine diseases globally, currently affecting approximately 828 million people—corresponding to a global prevalence of 14%. Its burden continues to rise, with projections estimating that 1.3 billion individuals will be living with diabetes by 2050 [1, 2]. Type 2 diabetes mellitus (T2DM) accounts for approximately 98% of all cases and is associated with a wide array of complications that significantly impact morbidity and mortality [3, 4]. Among these, diabetic neuropathy is one of the most common and debilitating microvascular complications [5], affecting up to 50% of patients with diabetes [6, 7].

Distal symmetric polyneuropathy (DSPN), the most common subtype, comprises approximately 75% of diabetic neuropathy cases. According to the American Diabetes Association, DSPN is defined as “the presence of symptoms and/or signs of peripheral nerve dysfunction in people with diabetes after the exclusion of other causes” [5]. It typically presents with distal, symmetric sensory loss, pain, or motor deficits in a length‐dependent “stocking–glove” pattern [8]. DSPN not only impairs quality of life but is also a major risk factor for diabetic foot ulcers, Charcot neuroarthropathy [9, 10], falls and fractures [11, 12], ultimately contributing to increased healthcare costs and mortality [5]. Despite its clinical importance, there is no curative treatment for DSPN, and current therapies are limited to controlling symptoms and slowing disease progression [5].

Hyperglycemia‐induced oxidative stress is a central mechanism in the pathogenesis of diabetic neuropathy [13]. Several metabolic pathways—including the polyol, hexosamine [14, 15], and advanced glycation end‐product pathways [16, 17]—converge to generate excessive reactive oxygen species (ROS), damaging peripheral nerves. Mitochondrial dysfunction and impaired axonal transport further exacerbate length‐dependent axonopathy [18, 19, 20].

Zinc, an essential trace element, plays a critical role in antioxidant defence [21], insulin metabolism [22, 23], and neuronal protection [24]. It is a structural constituent of numerous enzymes, including those involved in intracellular antioxidant defence [21], and stimulates metallothionein expression [24, 25, 26], which scavenges ROS. Zinc deficiency disrupts oxidative balance [21], may worsen hyperglycemia [27, 28], and has been implicated as a potential contributor to diabetic complications because of its antioxidant properties, suggesting that zinc deficiency may play a role in the pathogenesis of diabetic neuropathy [29, 30, 31]. In animal models, zinc supplementation ameliorates neuropathic symptoms and reduces oxidative stress [32]. Diabetic patients are particularly prone to zinc depletion due to hyperglycemia‐related urinary loss and poor absorption [33, 34, 35]. Limited studies in patients with diabetic polyneuropathy have shown that zinc supplementation can improve glycemic control, electrophysiological parameters of diabetic polyneuropathy, and markers of oxidative stress [36, 37].

Although animal and in vitro studies have strongly suggested a protective role for zinc against diabetic neuropathy [24, 25, 32, 38], clinical data remain limited and inconsistent. Few human studies have explored the associations between serum zinc levels and the presence and severity of DSPN, and the findings have been inconsistent [39, 40].

Given the limited clinical evidence and the high burden of DSPN, this study evaluated the associations between serum zinc levels and the presence of DSPN in patients with type 2 diabetes attending the internal medicine clinic of Shahid Beheshti Hospital in Hamadan, Iran.

## 2 Methods

### 2.1 Study Design and Setting

This nested case–control study was conducted at the internal medicine clinic of Shahid Beheshti Hospital in Hamadan, Iran, between December 2023 and March 2025. This study aimed to assess the associations between serum zinc levels and the presence of DSPN in patients with type 2 diabetes mellitus.

### 2.2 Study Population

During the study period, patients with T2DM who presented to the outpatient internal medicine clinic on days when the research team was present were screened for eligibility. A total of 143 patients were assessed. Of these, 42 did not meet the inclusion criteria, and 11 met at least one exclusion criterion. Finally, 90 eligible patients were consecutively enrolled in the study. Individuals were assigned to either the case group—patients diagnosed with DSPN—or the control group—patients with T2DM but without evidence of neuropathy (45 with diabetic peripheral neuropathy and 45 without neuropathy). Based on the study by Mona Hussein et al., assuming a two‐sided α of 0.05, a power of 80%, a mean difference in serum zinc levels of 6 μg/dL between groups, and a standard deviation of 10 μg/dL, the required sample size was estimated at 90 participants in total, with 45 participants per group.

The inclusion criteria were as follows: Diagnosis of T2DM for at least five years, age between 30 and 70 years, glomerular filtration rate (GFR) > 30 mL/min/1.73 m^2^, and HbA1c ≤ 10% (Both patients with controlled and uncontrolled type 2 diabetes were eligible for inclusion). Exclusion criteria included a history of type 1 diabetes; neuropathies of nondiabetic aetiology; concurrent thyroid, parathyroid, liver, or autoimmune disease; kidney disease other than diabetic nephropathy; active infections; pregnancy or lactation; use of zinc supplements or zinc‐depleting medications (e.g., PPIs for > 6 months, corticosteroids, diuretics, anticonvulsants); opioid or alcohol use; use of medications for neuropathic symptom relief or antioxidant therapy; Vitamin B12 deficiency; history of cerebrovascular disease or discopathies; recent use of anti‐inflammatory drugs; foot infections or wounds; cognitive or psychiatric disorders impairing cooperation; and refusal to participate or continue.

### 2.3 Diagnostic Criteria

The diagnosis of T2DM was confirmed based on the American Diabetes Association (ADA) criteria, whereby the presence of any one of the following is sufficient for diagnosis: A fasting plasma glucose ≥ 126 mg/dL, a 2‐h plasma glucose ≥ 200 mg/dL during an oral glucose tolerance test, a haemoglobin A1c ≥ 6.5%, or a random plasma glucose ≥ 200 mg/dL in the presence of classic symptoms of hyperglycemia. Screening for DSPN was performed via the Neuropathy Symptom Score (NSS) (Appendix 1) and the Neuropathy Disability Score (NDS) (Appendix 2). Patients with an NDS ≥ 6 or an NDS ≥ 3 in combination with an NSS ≥ 5 were classified as probable cases of DSPN. Those who screened positive were definitively diagnosed with DSPN by a relevant specialist based on the ADA criteria [5] for diabetic polyneuropathy: “A combination of typical symptomatology and symmetrical distal sensory loss or typical signs in the absence of symptoms (Appendix 3) in a patient with diabetes is highly suggestive of DSPN and may not require additional evaluation or referral.”

### 2.4 Data Collection and Measurements

Data collection involved structured interviews, clinical examinations, venous blood sampling, and review of medical records. Baseline and demographic information included age, sex, height, weight, body mass index (BMI), blood pressure, smoking status, time since diabetes diagnosis, insulin usage, HbA1c levels, blood urea nitrogen, serum creatinine, lipid profile (LDL‐ cholesterol, HDL‐ cholesterol, triglycerides), C‐reactive protein (CRP), comorbid conditions, and the use of antidiabetic medications and drugs that could confound the study results, including zinc supplements, zinc‐depleting medications (e.g., proton pump inhibitors for > 6 months, corticosteroids, diuretics, anticonvulsants), medications for neuropathic symptom relief, antioxidant and anti‐inflammatory drugs. The NSS and NDS were used to screen for DSPN. NSS scoring involves patient‐reported symptoms, with a maximum score of 9 (Appendix 1), and has demonstrated a sensitivity of 81.4% and specificity of 68.7% for detecting DSPN compared with nerve conduction studies [41, 42]. NDS scoring evaluates ankle reflexes, vibration, pinprick, and temperature sensation, with a total score ranging from 0 to 10 (Appendix 2) [41]. It has a sensitivity of 92.3% and specificity of 47.6%, with high interrater reliability (ICC ≥ 0.9) [42, 43].

Serum zinc levels were measured using a colourimetric single‐point photometric assay (Delta Darman Part, Iran). According to the manufacturer's analytical performance data, the assay demonstrates acceptable precision (intra‐assay CV 1.7%–2.8% and inter‐assay CV 1.8%–3.4%) and was validated by the manufacturer against the JaiCA/MC‐Reagent commercial zinc assay kit, showing a high correlation (*r* = 0.9947) and regression equation (Y = 0.8984 × + 8.8622) reported in the kit documentation. Venous blood samples were obtained from all participants, who were not fasting before sample collection, and stored at 2°C–8°C for a maximum of five days before analysis. The instruments used during the clinical assessments included a reflex hammer, 128 Hz tuning fork, neurotip, calibrated measuring tape, digital scale, metal tubes for thermal testing, and a calibrated aneroid sphygmomanometer.

### 2.5 Ethical Approval and Informed Consent

All participants provided written informed consent before enrollment. The Ethics Committee of Hamadan University of Medical Sciences approved this study (Approval ID: IR.UMSHA.REC.1402.607).

### 2.6 Statistical Analysis

Descriptive statistics were used to summarise the baseline characteristics. Continuous variables are reported as the means and standard deviations, and categorical variables are reported as frequencies and percentages. Independent samples *t*‐tests and chi‐square tests were used to compare the two groups.

To evaluate the independent association between serum zinc levels and the presence of DSPN while controlling for potential confounders, hierarchical binary logistic regression analysis was performed. In the first block, age, sex, and HbA1c were entered as fixed covariates to control for their potential confounding effects. In the second block, Variables with a *p*‐value < 0.2 in univariate comparisons, including BMI, insulin use, urea, GFR, and serum zinc level, were entered using the forward likelihood ratio stepwise method. Odds ratios (ORs) with 95% confidence intervals (CIs) were calculated. Model fit was assessed using the Omnibus test, Cox and Snell $R^{2}$, Nagelkerke $R^{2}$, and the Hosmer–Lemeshow goodness‐of‐fit test. A two‐sided p<0.05 were considered statistically significant. Multicollinearity among the independent variables was examined using variance inflation factors (VIF) and tolerance statistics obtained from a linear regression model using the Enter method. VIF values below 5 and tolerance values above 0.20 were considered indicative of acceptable levels of collinearity.

All the statistical analyses were performed via SPSS version 27, and a *p*‐value < 0.05 was considered to indicate statistical significance.

## 3 Results

A total of 90 patients with type 2 diabetes mellitus were enrolled, comprising 45 individuals with diabetic polyneuropathy (case group) and 45 without neuropathy (control group). The baseline and demographic characteristics of the two groups are summarised in Table 1. No statistically significant differences were observed between the groups in terms of sex distribution, age, duration of diabetes, serum creatinine, lipid profile (triglycerides, LDL‐ cholesterol, HDL‐ cholesterol), CRP, blood pressure, body mass index (BMI), diabetic nephropathy status, smoking, or insulin use (*p* > 0.05 for all). However, the HbA1c and blood urea levels were significantly greater in the case group (*p* = 0.008 and *p* = 0.045, respectively). Variables with a *p*‐value < 0.2 (serum zinc, HbA1c, urea, GFR, BMI, and insulin use), along with age and sex, were selected for adjustment in subsequent multivariable logistic regression analysis. The mean serum zinc level in the control group was 82.2 ± 16.9 μg/dL, whereas it was 70.6 ± 12 μg/dL in the case group. This difference was statistically significant (*p* < 0.001, Table 1), indicating significantly lower zinc levels in patients with diabetic polyneuropathy.

**TABLE 1:** Comparison of the baseline, demographic variables, and serum zinc levels between the case and control groups.

Variable		Control group	Case group	*p*‐value
Sex	Male, *n*(%) Female *n*(%)	13 (28.9%) 32 (71.1%)	10 (22.2%) 35 (77.8%)	0.46
Age (years)		9.6 ± 58.6	8.5 ± 58.6	0.97
Diabetes duration (months)		98.4 ± 127.1	64.1 ± 116.7	0.55
DPN duration (month)			5.3 ± 9.4	
Blood Urea (mg/dl), mean ± SD		7.7 ± 29.4	11.3 ± 33.6	0.045
Creatinine (mg/dl), mean ± SD		0.3 ± 0.9	0.3 ± 0.9	0.246
GFR (mL/min/1.73 m2), mean ± SD		18.7 ± 82.3	21.3 ± 75	0.085
HbA1c (%), mean ± SD		1.1 ± 7.6	1.3 ± 8.2	0.008
TG (mg/dl), mean ± SD		139.2 ± 183.2	93.7 ± 182.4	0.97
LDL‐ cholesterol (mg/dl), mean ± SD		25.3 ± 79.3	35.3 ± 79.7	0.95
HDL‐ cholesterol (mg/dl), mean ± SD		11.9 ± 42.5	14.7 ± 42.4	0.96
CRP (mg/l), mean ± SD		4.9 ± 2.8	4.6 ± 4.7	0.76
SBP (mmHg), mean ± SD		20.3 ± 130.2	18.8 ± 132.4	0.59
DBP (mmHg), mean ± SD		10.5 ± 82.8	18.2 ± 83.4	0.84
BMI (kg/m^2^), mean ± SD		4.7 ± 27.9	5.4 ± 29.5	0.14
Diabetic nephropathy, n(%)	Present Absent	13 (28.9%) 32 (71.1%)	9 (20%) 36 (80%)	0.32
Smoking, n(%)	Smoker Nonsmoker	4 (8.9%) 41 (91.1%)	5 (11.1%) 40 (88.9%)	0.99
Insulin use, n(%)	User Nonuser	9 (20%) 36 (80%)	15 (33.3%) 30 (66.7%)	0.15
Serum zinc level (μg/dl), mean ± SD		16.9 ± 82.2	12.0 ± 70.6	< 0.001

Abbreviations: DPN: diabetic polyneuropathy, GFR: glomerular filtration rate, TG: triglyceride, LDL: low‐density lipoprotein, HDL: high‐density lipoprotein, SBP: systolic blood pressure, DBP: diastolic blood pressure, BMI: body mass index.

Hierarchical binary logistic regression analysis was performed to examine the independent association between serum zinc levels and diabetic polyneuropathy after adjusting for potential confounders. In Block 1, age, sex, and HbA1c were entered as fixed covariates. In Block 2, BMI, insulin use, urea, eGFR, and serum zinc level were entered using a forward likelihood ratio stepwise procedure. Among these variables, only serum zinc level met the criteria for entry into the final model, while BMI, insulin use, urea, and eGFR did not significantly improve model fit and were therefore excluded. In the first block, HbA1c was the only variable significantly associated with diabetic polyneuropathy (B = 0.474, OR = 1.606, *p* = 0.011), while age and sex were not significant predictors. The inclusion of the second block significantly improved the model fit (likelihood ratio χ^2^ = 11.310, df = 1, *p* = 0.001). The overall final model was statistically significant (χ^2^ = 18.806, df = 4, *p* = 0.001). The model demonstrated acceptable explanatory power with a Nagelkerke R^2^ of 0.251. Model calibration was adequate, as indicated by a non‐significant Hosmer–Lemeshow goodness‐of‐fit test (*p* = 0.890). In the final model, serum zinc level entered the model as an independent predictor and was inversely associated with diabetic polyneuropathy (B = −0.056, OR = 0.946). This indicates that each 1 μg/dL increase in serum zinc level was associated with approximately a 5.4% reduction in the odds of diabetic polyneuropathy after adjustment for age, sex, and HbA1c (Table 2). HbA1c also remained significantly associated with diabetic polyneuropathy in the final model (B = 0.436, OR = 1.547, *p* = 0.027). This indicates that for each one‐unit increase in HbA1c, the odds of diabetic polyneuropathy increased by approximately 54.7%. Age and sex remained non‐significant in the final model. The classification accuracy of the model improved from 50.0% in the null model to 65.6% after inclusion of the covariates. To evaluate potential multicollinearity among the independent variables included in the hierarchical model, variance inflation factors (VIF) were calculated using a linear regression procedure with the Enter method. All variables demonstrated acceptable VIF values ranging from 1.081 to 1.347, with tolerance values ranging from 0.742 to 0.925, indicating the absence of problematic multicollinearity. Pearson's correlation between HbA1c and the serum zinc level across the entire study population was negative (r = −0.160) but not statistically significant (*p* > 0.05).

**TABLE 2:** Hierarchical binary logistic regression analysis of factors associated with diabetic polyneuropathy.

Index Variable							95% Cl for odds ratio	
	B	S.E	Wald	df	*p*‐value	Exp(B) (odds ratio)	Lower	Upper
Block 1								
Age	−0.009	0.025	0.128	1	0.721	0.991	0.945	1.040
Sex	0.224	0.506	0.195	1	0.658	1.251	0.464	3.374
HbA1c	0.474	0.187	6.446	1	0.011	1.606	1.114	2.316
Constent	−3.393	1.919	3.126	1	0.077	0.034		
Block 2 (final Model)							SS	
Age	−0.009	0.026	0.106	1	0.745	0.991	0.941	1.044
Sex	−0.042	0.537	0.006	1	0.937	0.958	0.335	2.743
HbA1c	0.436	0.197	4.881	1	0.027	1.547	1.050	2.277
Serum Zinc	−0.056	0.019	8.818	1	0.003	0.946	0.912	0.981
Constant	−1.299	2.474	0.276	1	0.599	3.667		

*Note:* Values are presented as regression coefficient (B), standard error (SE), Wald statistic, odds ratio (OR), and 95% confidence interval (CI). In Block 1, age, sex, and HbA1c were entered as fixed covariates. In Block 2, serum zinc level was added to the hierarchical model. Variables not retained in the final model are not shown. OR > 1 indicates increased odds of DSPN, whereas OR < 1 indicates decreased odds. Sex(1) indicates that the dependent variable was the presence of DSPN (yes/no).

## 4 Discussion

Diabetic neuropathy is the most common chronic complication of diabetes, with distal symmetric sensorimotor polyneuropathy (DSPN) accounting for approximately 75% of cases [5]. Zinc deficiency has been extensively investigated among the mechanisms proposed in diabetic neuropathy's pathogenesis because of its role in oxidative stress regulation, insulin metabolism, and neuronal function.

In the present nested case–control study, patients with DSPN had significantly lower serum zinc levels than diabetic controls without neuropathy did. After adjustment for potential confounding factors, serum zinc level remained significantly associated with diabetic distal symmetric polyneuropathy (DSPN). In the multivariable logistic regression analysis, higher serum zinc levels were independently associated with lower odds of DSPN (OR = 0.946), indicating that each 1 μg/dL increase in serum zinc level was associated with approximately a 5.4% reduction in the odds of DSPN. These findings suggest that lower serum zinc levels may be independently linked to the presence of DSPN in patients with type 2 diabetes.

The observed reduction in serum zinc concentrations among patients with DSPN can be attributed to a complex, bidirectional pathophysiological relationship. On one hand, zinc deficiency directly exacerbates the mechanisms leading to neural injury. As a critical cofactor for antioxidant enzymes such as superoxide dismutase and metallothionein, zinc is essential for neutralising reactive oxygen species (ROS) [44]. In the context of chronic hyperglycemia, ROS accumulation leads to mitochondrial damage and disrupts their axonal transport, triggering the length‐dependent axonal degeneration seen in DSPN [13, 18, 19, 20]. Furthermore, zinc is integral to insulin storage, secretion, and structural integrity; its deficiency is associated with increased insulin resistance and impaired glycemic control—both of which are key drivers of diabetic complications [22, 23]. Given that zinc also supports neuronal survival and synaptic plasticity, its depletion directly compromises nerve integrity and renders the peripheral nervous system more vulnerable to injury [24, 45].

On the other hand, poorer glycemic control in patients with DSPN may itself account for the lower serum zinc concentrations observed in this group. In our study, higher HbA1c levels were independently associated with increased odds of neuropathy (OR = 1.547), indicating that patients with DSPN had worse metabolic control. Each 1% increase in HbA1c was associated with approximately a 55% increase in the odds of neuropathy, underscoring the strong impact of poor glycemic regulation on neural injury. Chronic hyperglycemia is a well‐established contributor to diabetic complications and may also promote zinc depletion. In this context, sustained poor glycemic control can lead to increased urinary zinc excretion (hyperzincuria), thereby reducing circulating zinc levels [46, 47, 48]. Thus, the lower serum zinc concentrations observed in patients with neuropathy may, at least in part, reflect a metabolic consequence of inadequate glycemic control rather than solely a primary etiological factor. The study by Luo et al. [39], also reported lower serum zinc levels in diabetic patients with neuropathy than those without, which is consistent with our findings. However, after adjusting for confounding variables, they found no independent association between serum zinc and neuropathy in their logistic regression model. A notable methodological limitation of Luo et al.'s study was the lack of clearly reported exclusion criteria, which may have allowed the inclusion of patients with neuropathy due to nondiabetic causes. Additionally, unlike our study, which used ADA clinical criteria, they relied on a combination of physical examination and somatosensory evoked potentials (SSEPs), which may identify different patient subsets.

In contrast to our findings, the study byMona Hussein et al. [40], reported no significant difference in serum zinc levels between diabetic patients with and without neuropathy. The diabetic sample size was comparable to ours (40 patients with neuropathy and 40 without neuropathy). Moreover, they did not adjust for key potential confounders such as lipid profile, urea, creatinine, BMI, smoking status, eGFR, blood pressure, and the presence of diabetic nephropathy—factors that were adjusted for in our analysis. They also employed nerve conduction studies for diagnosis, which adds objectivity, but their analysis lacked multivariable control. In contrast to their findings—where no significant difference in HbA1c was observed between patients with and without neuropathy—we found that patients with neuropathy had significantly higher HbA1c levels.

Unlike the findings of Mona Hussein et al. [40], however, and in line with the findings of Akhuemokhan et al. [49], no significant correlation was found between serum zinc levels and HbA1c in all diabetic patients. Although poor glycemic control may exacerbate oxidative stress and promote zinc loss via hyperzincuria, the complex interplay between zinc and glycemic indices may be influenced by additional metabolic, dietary, or genetic factors not fully captured in this study.

Regarding renal function, elevated serum urea in the neuropathy group likely reflects comorbid diabetic nephropathy. Prior studies have shown that a reduced GFR and the accumulation of uremic toxins, such as urea, can contribute to neural injury via mechanisms similar to those observed in DSPN [50, 51, 52]. Nonetheless, serum urea was not independently associated with neuropathy in our adjusted analysis, reinforcing the distinct role of zinc.

Our results have potential clinical implications. The serum zinc level may be a useful biomarker for identifying patients at increased risk for DSPN. If validated in larger prospective studies, zinc supplementation could emerge as a complementary strategy for preventing or slowing the progression of diabetic neuropathy. Additionally, incorporating serum zinc assessment into neuropathy screening protocols may improve early detection and personalise treatment approaches.

This study has several limitations. The sample size was relatively small and drawn from a single centre, which may limit generalisability. The nested case–control design prevents inference of causality. Although major confounders were adjusted for, residual confounding from unmeasured factors—such as dietary zinc intake or the status of other micronutrients—cannot be excluded. In addition, serum zinc concentrations are homeostatically regulated and may not decline in cases of marginal zinc intake; therefore, serum zinc alone may not fully reflect individual zinc status [53]. The absence of electrodiagnostic testing limits diagnostic precision. Although the colourimetric method is analytically precise, previous studies have reported variable agreement with reference methods such as AAS and ICP‐MS [54, 55]. Since we did not perform cross‐validation with these gold standards, our results should be interpreted with this limitation in mind.

In light of these findings, future studies should adopt prospective, multicenter designs with larger sample sizes to improve generalisability. Incorporating objective electrophysiological tools, such as nerve conduction studies, would increase the accuracy of neuropathy assessment. Moreover, a detailed evaluation of dietary zinc intake and other micronutrient statuses is essential to minimise residual confounding. Randomised controlled trials are needed to assess whether zinc supplementation reduces neuropathy severity or delays its progression. Further investigations into the molecular mechanisms and longitudinal patterns of zinc variation may also provide deeper insights into the pathophysiology of diabetic neuropathy.

## 5 Conclusions

This study revealed that patients with type 2 diabetes mellitus (T2DM) and symmetric distal diabetic polyneuropathy had significantly lower serum zinc levels than patients with T2D without neuropathy. The serum zinc concentration was independently and inversely associated with the presence of diabetic polyneuropathy, indicating that higher zinc levels may be associated with a lower likelihood of developing this complication. These results underscore the potential role of zinc deficiency in the pathogenesis and progression of diabetic neuropathy in T2DM patients.

## Author Contributions

**Firozeh Sajedi:** conceptualization, supervision, methodology, validation. **Hosseinali Mohammadi:** writing – original draft, conceptualization, methodology, investigation. **Younes Mohammadi:** data curation, validation, formal analysis.

## Funding

This work was supported by the Hamadan University of Medical Sciences (140209147896).

## Ethics Statement

This study was conducted in accordance with the ethical standards of the Declaration of Helsinki and approved by the Ethics Committee of Hamadan University of Medical Sciences. The protocol was reviewed and approved under reference number IR.UMSHA.REC.1402.607. Written informed consent was obtained from all participants prior to enrolment.

## Consent

The authors have nothing to report.

## Conflicts of Interest

The authors declare no conflicts of interest.

## Data Availability

The data that support the findings of this study are available on request from the corresponding author. The data are not publicly available due to privacy or ethical restrictions.

## Appendix A

### Appendix 1 Neuropathy Symptom Score (NSS) Questionnaire (41)

Instructions: Please answer the following questions on the basis of your usual symptoms. For each item, choose the option that best describes your experience. The total score will help us assess the severity of your neuropathic symptoms.

1. What kind of discomfort or pain do you usually feel in your legs? (Choose the most prominent symptom.)

☐ Burning, numbness, or tingling — 2 points

☐ Fatigue, cramping, or aching — 1 point

☐ No symptoms — 0 points

2. Where do you usually feel these symptoms? (Choose the primary location.)

☐ Feet — 2 points

☐ Calves — 1 point

☐ Elsewhere — 0 points

3. When do you notice the symptoms the most?

☐ At night (nocturnal exacerbation) — 2 points

☐ Both during the day and night — 1 point

☐ Only during the day — 0 points

4. Have your symptoms ever woken you from sleep?

☐ Yes — 1 point

☐ No — 0 points

5. What makes your symptoms better? (Choose the most effective action.)

☐ Walking — 2 points

☐ Standing — 1 point

☐ Sitting or lying down — 0 points

Total NSS score: _____/9

### Appendix 2 Neuropathy Disability Scale (NDS) Assessment Form (41)

Instructions: Assess the following modalities on both feet. The patients were asked to keep their eyes closed during sensory testing. Each item is scored per side. The total score is calculated on the basis of the criteria below. The maximum possible score is 10.

**1. Ankle Reflex**

☐ Normal—0 points (right foot)

☐ Normal—0 points (left Foot)

☐ Present with reinforcement—1 point (right foot)

☐ Present with reinforcement—1 point (Left Foot)

☐ Absent—2 points (right foot)

☐ Absent—2 points (left Foot)

**2. Vibration Senses (at the great toe)**

☐ Present—0 points (right foot)

☐ Present—0 points (left Foot)

☐ Reduced/Absent—1 point (right foot)

☐ Reduced/Absent—1 point (left Foot)

**3. Pin‐prick sensation (at the great toe)**

☐ Present—0 points (right foot)

☐ Present—0 points (left Foot)

☐ Reduced/Absent—1 point (right foot)

☐ Reduced/Absent—1 point (left Foot)

**4. Temperature sensation (cold tuning fork at the great toe)**

☐ Present—0 points (right foot)

☐ Present—0 points (left Foot)

☐ Reduced/Absent—1 point (right foot)

☐ Reduced/Absent—1 point (left Foot)

Total NDS score: _____/10

### Appendix 3 Symptoms and Signs of Dspn (5)

**Table:**

	Large myelinated nerve fibres	Small myelinated nerve fibres
Function	Pressure, balance	Nociception, protective sensation
Symptoms^a^	Numbness, tingling, poor balance	Pain: Burning, electric shocks, stabbing
Examination (clinically diagnostic)^b^	Ankle reflexes: Reduced/absent Vibration perception: Reduced/absent 10‐g monofilament: Reduced/absent Proprioception: Reduced/absent	Thermal (cold/hot) discrimination: Reduced/absent^b^ Pinprick sensation: Reduced/absent^b^

^a^ To document the presence of symptoms for diagnosis.

^b^ Documented in symmetrical, distal to proximal pattern.
